# Implementation of portable head CT imaging in patients with severe acute brain injury in a French ICU: a prospective before–after design pilot study

**DOI:** 10.1038/s41598-022-25263-6

**Published:** 2022-12-02

**Authors:** Launey Yoann, Mycinski Clément, Eugène François, Bellec Elise, Serpolay Hubert, Ferré Jean-Christophe, Seguin Philippe, Gauvrit Jean-Yves

**Affiliations:** 1 Critical Care Unit, Department of Anaesthesia, Critical Care and Perioperative Medicine, Centre Hospitalier Universitaire de Rennes, 2 Rue Henri Le Guilloux, 35033 Rennes, France; 2 University of Rennes, 2, Rue Henri Le Guilloux, 35033 Rennes, France; 3 Department of Neuroradiology, Centre Hospitalier Universitaire de Rennes, 2 Rue Henri Le Guilloux, 35033 Rennes, France; 4 Radiation Protection Unit, Department of Biomedical Engineering, Centre Hospitalier Universitaire de Rennes, 2 Rue Henri Le Guilloux, 35033 Rennes, France; 5 Head office of the Department of Biomedical Engineering, Centre Hospitalier Universitaire de Rennes, 2 Rue Henri Le Guilloux, 35033 Rennes, France

**Keywords:** Neurology, Neurological disorders, Brain injuries

## Abstract

Head-CT-scanning is a cornerstone procedure during the management of patients admitted for acute brain injury (ABI) in intensive care unit (ICU). But intrahospital transfer for these procedure is known to increase the rate of severe adverse events potentially worsening the brain injuries. Portable head-CT (pCTH) may facilitate pCTH performance in safer conditions for the patients avoiding transfer out of the ICU. To evaluate the safety and the time duration required to use a portable head CT (pCTH) scanner in the intensive care unit (ICU) in the French healthcare system in ICU patients admitted for acute brain injury, we prospectively included all ICU-patients admitted for severe ABI over a 2-year period following before–after design. As the main outcome, we compared the time required to perform a scan with pCTH to that with conventional head CT (cCTH) and reported adverse events and reactions. In total, forty-six patients were included and finally, 41 patients were analyzed (21 in the pCTH group and 20 in the cCTH group). The median (interquartile) time required to perform a scan with pCTH was 28 (23–48) minutes compared to 30 (25–36) minutes with cCTH (p = 0.825). The duration time required to perform a pCTH was similar to that with cCTH in an ICU of the French healthcare system without significant difference in adverse events reactions.

## Introduction

Head computed tomography (CTH) imaging is a cornerstone procedure in early management of acute brain injury (ABI) patients admitted to the intensive care unit (ICU)^1^. It is frequently repeated to assess the evolution of brain lesions and their complications in clinical situations where neurological examinations may be insufficient for clinical assessment^2^. CTH performance commonly requires moving the ICU patient to the radiology department during a critical clinical period in which the patient may be unstable and potentially exposed to complications such as intracranial hypertension (ICH) and respiratory or haemodynamic instability^3,4^. Indeed, intrahospital transfer (IHT) of ICU patients is known to increase the rate of complications (ICH, hypotension, hypoxemia, and ventilator disconnection or ventilator asynchrony) that may worsen the prognosis of the patient and increase the length of ICU stay^5^. These complications associated with IHT are frequent and may occur in up to 80% of patients needing IHT^6,7^. Moreover, to ensure the safety of the patient during IHT, a dedicated team is required, including a doctor, a nurse and a hospital porter, which may reduce the ability of these individuals to care for other ICU patients, according to the local healthcare system organization. In this context, portable computed tomography for the head (pCTH) may be useful. It is a technique already used in different countries^8^ and can reduce the duration of CTH compared to conventional CTH (cCTH) and consequently the duration of patient exposure to adverse events, especially ICH^9^. It may also reduce the cost of the procedure by decreasing the need for a large ICU team and freeing up time slots for conventional CTH. A study at the Massachusetts General Hospital, USA, demonstrated that pCTH could significantly reduce the delay to imaging access during stroke compared to cCTH (39.0 ± 5.1 min versus 17.0 ± 2.7 min, respectively)^10^. Another American study estimated a financial gain of 2 million dollars over 5 years when using pCTH compared to cCTH^11^. However, the healthcare system organization may influence the implementation and efficiency of such a tool, and replication of these results in a different healthcare system, such as that in France, has not been attempted. Indeed, to date, none of the French ICUs has explored the feasibility of using pCTH in clinical ICU situations. The main objective of our study was, to assess the time duration of the pCTH procedure and to report adverse events and reactions compared to the cCTH procedure in ICU patients admitted for ABI.

## Results

**Figure 1:**
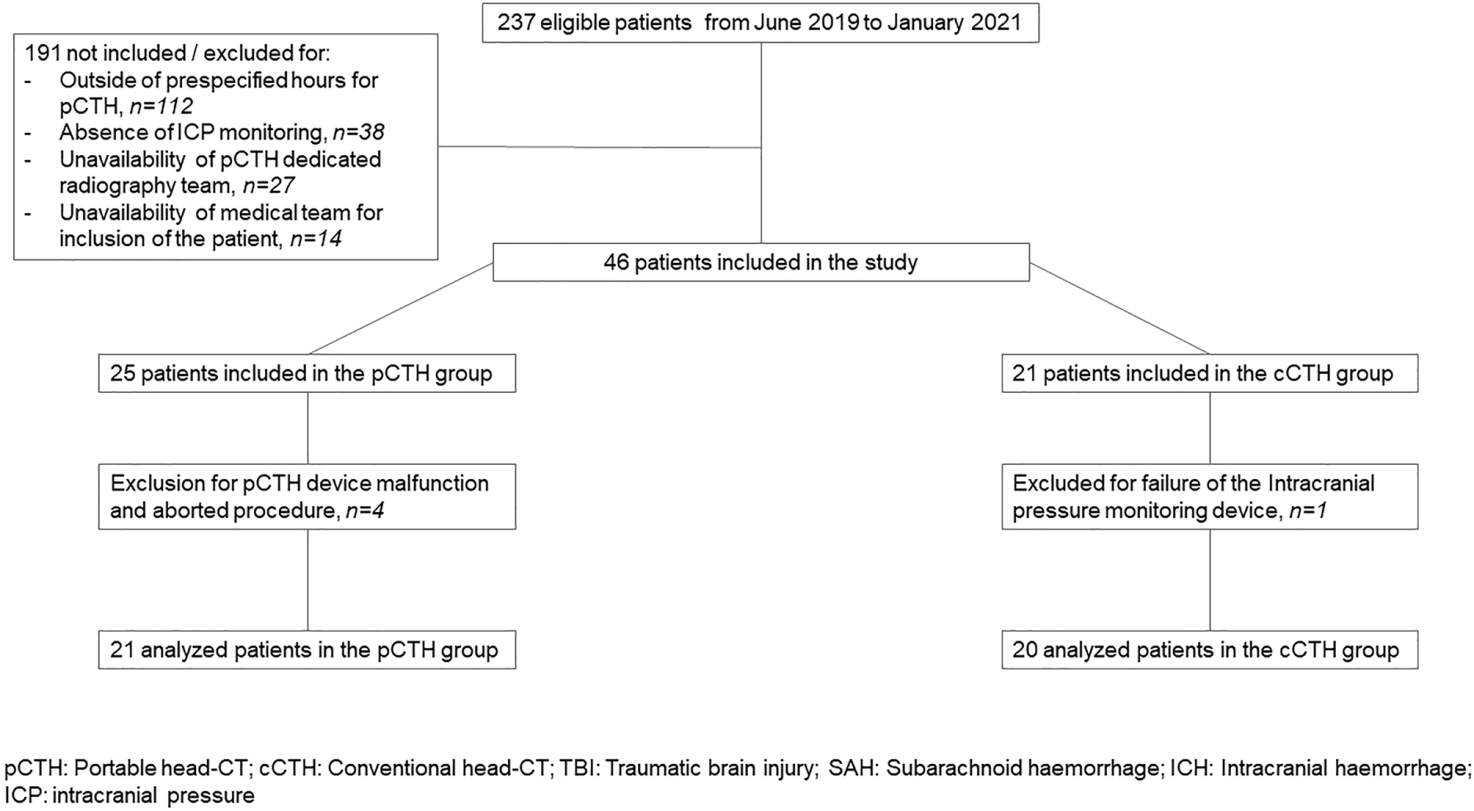
Flowchart of the study.

Forty-six patients were included in the study: 25 in the pCTH group and 21 in the cCTH group. Four patients were excluded for reasons of pCTH device malfunction (n = 3), procedure interruption (n = 1) and one patient excluded in the cCTH group due to ICP monitoring failure. Finally, 41 patients were analysed: 21 patients in the pCTH group and 20 in the cCTH group were analysed (Fig. 1). Demographics and clinical data are reported in Table 1. We identified 4 high-risk patients in the pCTH group and 6 high-risk patients in the cCTH group.

**Table 1:** Demographic and clinical characteristics.

	Total	pCTH group	cCTH group
	n = 41	n = 21	n = 20
Age, *years*	54 (38–66)	59 (54–66)	40 (30–62)
Sex-ratio (f/m)	0,52 (14/27)	0,46 (7/15)	0,66 (8/12)
Weight, *kg*	74 (64–84)	75,0 (68,5–87,5)	69,5 (63,4–81,4)
Height, *cm*	176 (169–180)	173 (168–176)	179 (173–183)
Body mass index, kg/m^2^	24 (21–27)	25 (23–29)	23 (20–25)
**Reason for ICU admission**			
TBI	23	11	12
SAH	1	4	6
Acute subdural haematoma	1	1	0
Haemorrhagic stroke	5	4	1
Ischaemic stroke	2	1	1
GCS score at admission	6 (5–10)	8 (5—14)	6 (5–7)
**Reason for CTH**^**$**^			
Control of EVD location	2	1	1
Haematoma assessment	1	1	0
Control of ischaemia 24 h after stroke	1	0	1
Postoperative neurosurgical control	6	3	3
Control, 24 h–48 h after TBI	15	8	7
Intracranial hypertension	12	5	7
Hydrocephalus screening	2	2	0
Intracranial Ischaemia/bleeding screening	1	1	1
**Outcomes**			
In-ICU mortality, n	15	9	6
GOS at 3 months^§^			
*GOS 1*	18	9	9
*GOS 2*	1	0	1
*GOS 3*	6	6	0
*GOS 4*	10	4	6
*GOS 5*	6	2	3
*NA*	1	0	1

*Data are expressed as the median (interquartile) or n.*

*pCTH* portable head CT, *cCTH* conventional head CT, *GOS* Glasgow Outcome Scale, *ICU* intensive care unit, *TBI* traumatic brain injury, *SAH* subarachnoid haemorrhage, *GCS* Glasgow Coma Scale, *EVD* external ventricular drainage.

^§^one missing patient follow-up in cCTH group.

The main primary endpoint, the median (interquartile) time required to perform pCTH, was not significantly reduced compared to cCTH: 28 (23–48) minutes vs 30 (25–36) minutes, respectively (p = 0.825). Within the pCTH group, we identified a significantly longer duration for the first 8 pCTH procedures compared to the 13 following procedures: 53 min (37–59) vs 25 min (16–27) (p = 0.002).

**Figure 2:**
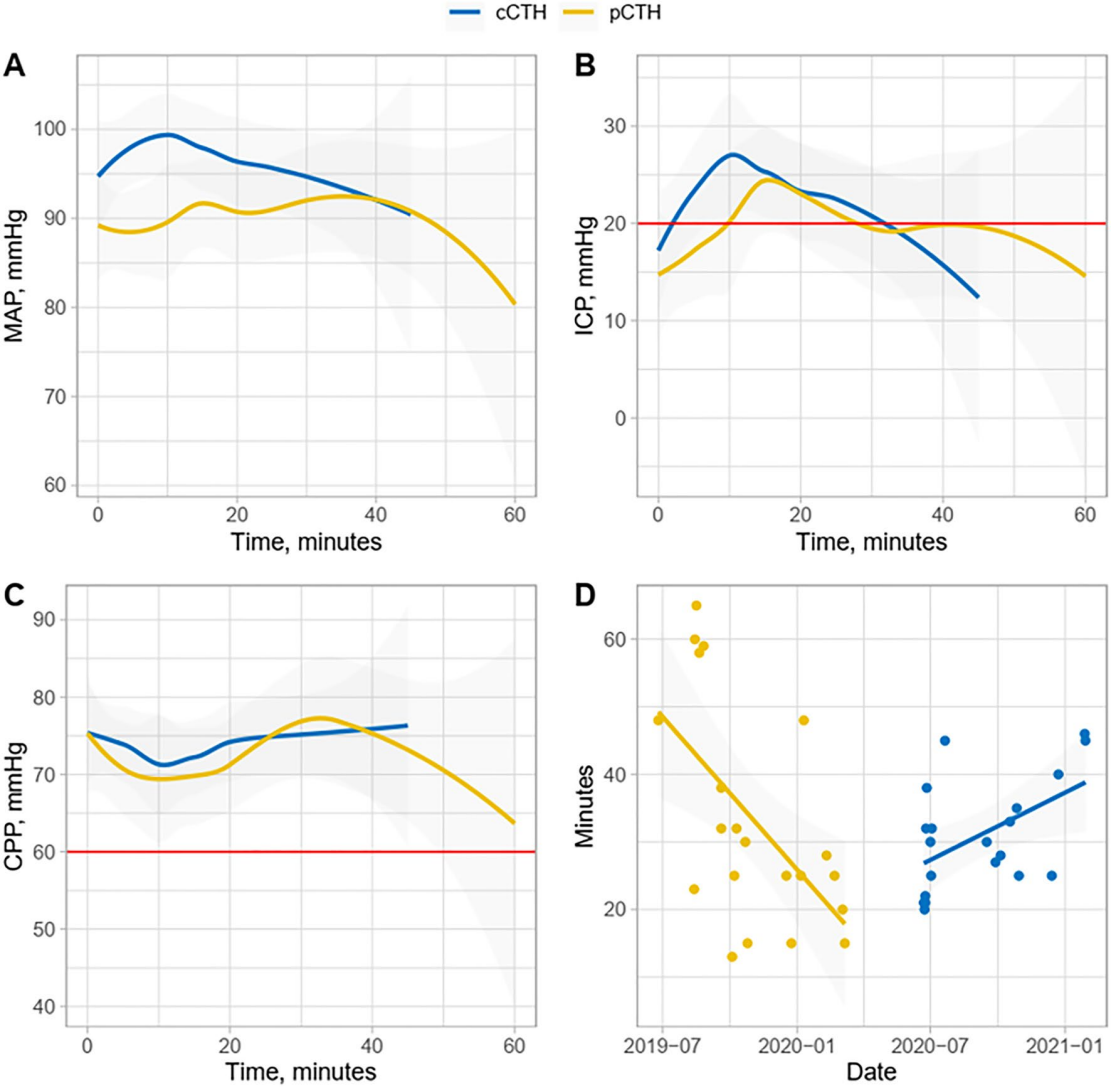
Temporal pattern of (**A**) mean arterial blood pressure (MAP), (**B**) intracranial pressure (ICP) and (**C**) cerebral perfusion pressure (CPP) during CT scanning with conventional head CT (cCTH, blue) and portable head CT (pCTH, yellow) and (**D**) individual scanning duration across the study. The blue and yellow lines in (**D**) represent the linear regression lines for each group.

Considering the secondary endpoints, especially adverse reactions, no major significant differences were observed between the 2 groups (Table 2). Disconnection of the ventilator was far less frequent in the pCTH group (1/21) compared to the cCTH group (6/20), although the difference did not reach statistical significance. The ICP and CPP patterns in each group were similar, with an insignificant trend towards higher CPP in the pCTH group (p = 0.160 and 0.358, respectively) (Fig. 2). We also observed a trend in greater need to increase the norepinephrine infusion rate in the cCTH group than in the pCTH group. No arrhythmias episodes were observed during CTH. One episode of SpO2 < 95% lasting 14 min were reported in pCTH group. The number of ventilator-acquired pneumonia (VAP) cases was not significantly different: 7 in the pCTH group versus 8 in the cCTH group. Similarly, we found only one central nervous system infection in a patient with EVD (pCTH group). The in-ICU mortality rate was not significantly different between the groups (9/21 in the pCTH group versus 6/20 in the cCTH group, p = 0.398). At 3 months, no significant difference were observed in outcomes based on Glasgow outcome scale (p = 0.539).

**Table 2:** Events observed during scanning (technical events and clinical adverse reactions).

	pCTH, *n* = *21*	cCTH, *n* = *20*	P
**Clinical, *n***	28	74	0.395
Mandatory ventilator disconnection	0	40	
Unexpected ventilator disconnection	1	6	
Noisy examination and prolonged supine position	1	0	
Switch from pressure support ventilation mode to controlled volume ventilation mode	4	0	
ICP > 20 mmHg	10	10	
ICP > 30 mmHg	6	8	
CPP < 60 mmHg	5	4	
SABP < 110 mmHg	0	1	
Increase in norepinephrine infusion rate	1	5	
Arrythmias	0	0	
Episodes of SpO2 < 95%	1	0	
Arterial or central line disconnection	0	0	
**Technical/organization failures, *n***	7	0	–
Procedure failure for low-battery device	1	–	–
Procedure abortion for simultaneous trauma centre admission	1	–	–
Built-in computer start-up failure	2	–	–
Image acquisition failure due to security stop system problem	1	–	–
Suspended acquisition due to urgent critical patient condition in adjacent ICU room	1	–	–
Procedure abortion due to movement of the pCTH system	1	–	–

*pCTH* portable head CT, *cCTH* conventional head CT, *ICU* intensive care unit, *ICP* intracranial pressure, *CPP* cerebral perfusion pressure, *SABP* systolic arterial blood pressure.

We observed seven failure events due to software or hardware malfunctions during the pCTH procedure during the study period (Table 2). Noteworthy, 3 patients of the pCTH group required an additional CT-scan using cCTH due to failure of the pCTH device. No additional CT-scan was required for poor quality images in pCTH group.

Regarding the involvement of staff in performing CTH, most of the procedures required a team including at least a physician, a nurse, a care assistant and a radiographer. Two radiographers were needed for most of the pCTH procedures (detailed in Table 3).

**Table 3:** Staff attendance during intrahospital transport for CTH.

Staff category	pCTH, *n* = *21*				cCTH, *n* = *20*			
	Absent	n = 1	n = 2	n = 3	Absent	n = 1	n = 2	n = 3
Radiographer	0	1	17	4	0	0	19	1
Nurse	0	19	2	0	0	18	2	0
Doctor	3	17	1	0	0	20	0	0
Care-assistant	2	14	5	0	2	18	0	0

*pCTH* portable head CT, *cCTH* conventional head CT.

## Discussion

In this study, we demonstrated that compared with cCTH, pCTH with a Ceretom^®^ device was performed in our ICU without reducing the time required for examination. We did not find significant difference in the number of adverse reactions between groups.

### Duration of pCTH

Several studies have previously reported the feasibility of pCTH with a CereTom portable CT scanner to generate satisfactory clinical images at acceptable patient doses^8^ and in ICU patients in the North American and Scandinavian healthcare systems^9,14,15^. In Ohio, USA, Masaryk et al. demonstrated, in a large cohort of 502 pCTH examinations in ICU patients over 4 months, that pCTH was faster than conventional imaging, as the mean procedure duration was 18 min compared to 50 min^11^. Unfortunately, standard deviations were not reported in this study. Similarly, using a different pCTH device model, a Swedish study reported a globally decreased scanning duration in the most severe neuro-ICU patients: 37 ± 12 min (range 20–60 min) for pCTH compared with 79 ± 36 min for cCTH (range 20–225 min), p-value < 0.05^9^. In our study, cCTH is easily accessible and consequently the mean procedure time duration for cCTH was shorter than the durations reported in previous studies, which may explain the absence of difference for procedure duration between the two groups.

### Safety of pCTH

Moreover, regarding the safety of pCTH, although systemic haemodynamics did not seem to be altered in either group, we observed a trend in higher requirement for vasopressors in the cCTH group than in the pCTH group and a trend towards a higher rate of ventilator disconnection. Regarding cerebral haemodynamics, MAP and CPP levels were similar in the two groups. This is consistent with the study from Peace et al., who showed that in 57 ICU patients, pCTH had little effect on ICP (mean ICP 14.1 ± 6.6 mm Hg, p = 0.85) and CPP (mean CPP 81.0 ± 19.8 mm Hg, p = 0.59)^16^. Nevertheless, several studies demonstrated that IHT could lead to severe adverse reactions, potentially jeopardizing patient safety^3,17^. Especially in acute brain injury ICU patients, secondary insults occurred significantly more frequently (in 52% of patients) during transport, whereas secondary insults were observed in 13% of patients before and after IHT^4^. In line with these data, in Sweden, the feasibility and safety of mobile CT scanners in the neuro-ICU were compared to cCTH^9^. In that study, medical complications or technical mishaps occurred during cCTH scanning in 23% of patients, whereas they dropped dramatically to 4.3% during pCTH scanning^9^. The most common adverse reactions during cCTH were blood pressure changes, desaturation and ventilator-related mishaps, and increases in ICP. In our study, although not statistically significant, unexpected disconnection of the ventilator was far less frequent in the pCTH, but as a secondary outcome, the power was too low to conclude on these adverse reactions (estimated power: 60%). In addition, planned ventilator disconnection was mandatory during cCTH procedure, as the change of ventilator device was required to warrant IHT. Consequently, the total number of ventilator disconnections (mandatory and unexpected) were more frequent in cCTH group. Finally, we identified 6 high-risk patients in the cCTH group who might have benefited from pCTH.

### Staff workload

During transport for cCTH, a higher ratio of nurses and accompaniers may be required for the most severely critically ill patients, a request that may be attenuated by pCTH. Indeed, Gunnarson et al. estimated a reduction in total nursing time of 145 min in higher-risk patients and of 64 min in medium-risk patients when using pCTH compared to cCTH^9^. Unfortunately, our study was not designed for a medico-economic evaluation of our staff organisation during CTH. We also observed the need for 2 radiographers during pCTH. An explanation could be the lack of pCTH training of radiographers, which may have increased the pCTH scanning time. To support this hypothesis, we observed a faster pCTH scanning duration after 8 CT scans, which may reflect a dedicated time needed to take control of the device. Conversely, we also identified an increase of scanning time in the late phase of cCTH group. One explanation might be the trend of more severe patients in the cCTH group as assessed by the Coma Glasgow scale. While, the difference on this parameter was not significant between the 2 groups, we reported higher need of catecholamines increase and higher number of ICP > 30 mmHg episodes which, by itself, may reflect a greater severity of the cCTH group.

### Limitations and strengths

This study suffers from several limitations. First, despite its prospective design, it is an observational pilot study with a small sample of patients. Second, the use of pCTH may have been not optimal because of the short training period for radiographers on the new device and consequently their lack of experience. Third, unfortunately, we had to cope with obstacles to pCTH, including that it requires a heavy non-motorized device that may have hampered the user-friendly aspect of this tool at bedside and made the duration of the examination longer. We also had to address pCTH device malfunctions (hardware and software). Additionally, for logistics reasons, especially trained radiographer availability, we had to dedicate a time slot for pCTH scanning, i.e., during the afternoon of working days. Fourth, we cannot exclude the possible impact of the COVID-19 pandemic despite trying to maintain the routine ICU procedure for CTH transport and performance. Fifth, we only performed pCTH without contrast. The quality of images was sufficient to answer to the clinical questions but the results might slightly differ if we had addressed this issue for pCTH with contrast.

Nevertheless, this is the first study evaluating the use of pCTH in the ICU within the French healthcare system. This pilot study was also a real-life study, as we encountered multiple device malfunctions in the context of the COVID-19 pandemic. Finally, our results differ slightly from those of previous studies published in other countries. Two explanations may account for this: (i) Structurally, our cCTH, which is positioned only one floor below our ICU and consequently easily accessible with no time constraints for ICU patients, does not remove the higher risk of intrahospital transport for these patients; (ii) the short period of training on pCTH use may have altered the benefit of this tool. It is possible that a longer duration of training could shorten the procedure time.

## Conclusion

This prospective observational pilot study demonstrated that the time duration for pCTH imaging in an ICU from a single French University Hospital was not significantly shorter than cCTH procedure. In addition, we found no increase in adverse reactions in the pCTH group compared to the cCTH group. Although there were fewer ventilator disconnections for pCTH procedures, our study lacked of power. These results encourage the use of pCTH at bedside in ICU for the most critically ill ABI patients, but additional prospective randomized studies are required in the French ICUs to confirm the results observed in the North American healthcare system and to assess accurately the benefit on adverse reactions between these two CT-scan modalities.

## Methods

This single-centre prospective observational study evaluated the time duration to complete the procedure of using pCTH compared to conventional CTH in an ICU of a French university hospital. The study used a before-after design to compare a group imaged using cCTH to a group imaged using pCTH. The before period (pCTH) was from June 2019 to March 2020, and the after period (cCTH) was from March 2020 to January 2021.

**Figure 3:**
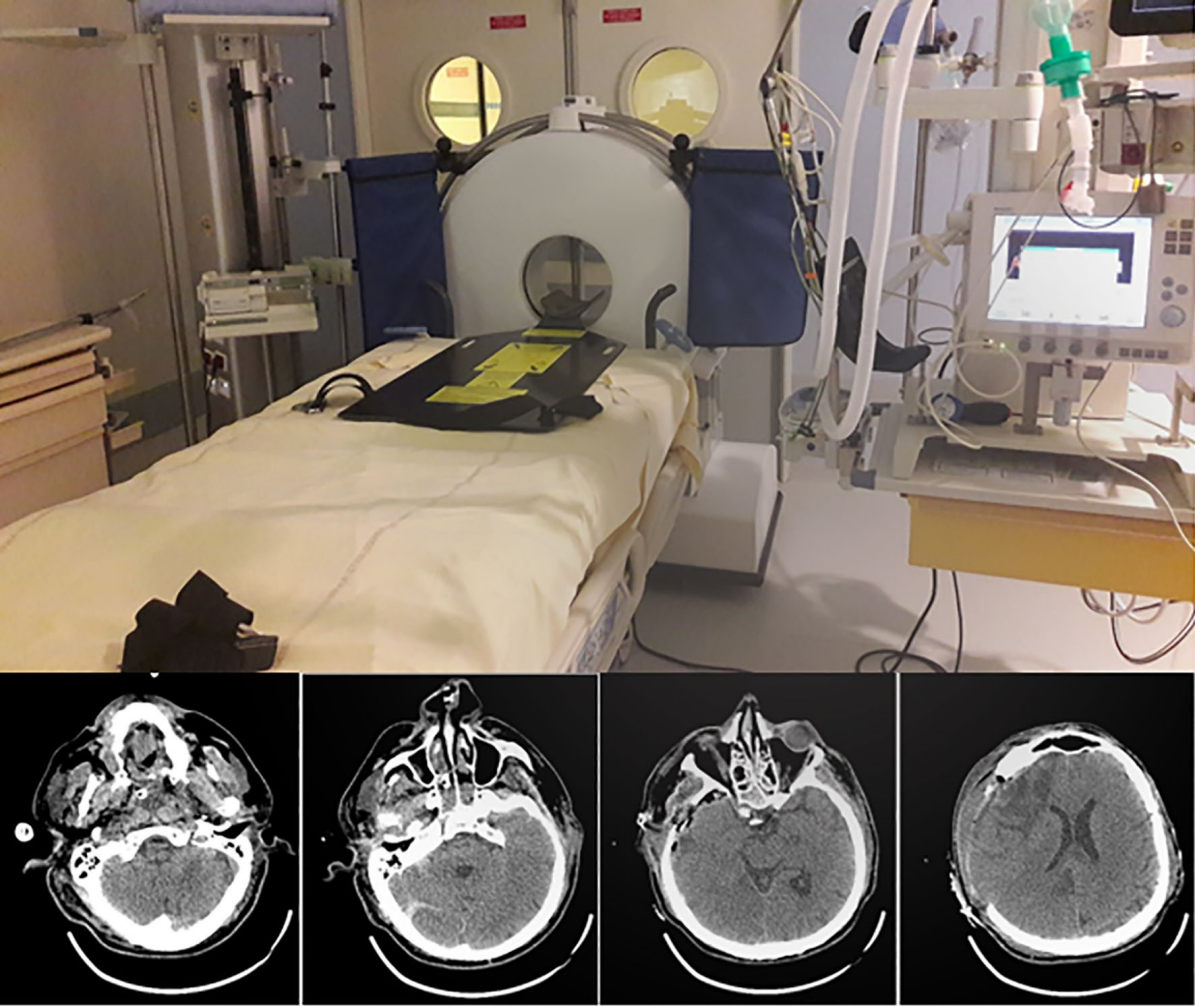
Position of the Ceretom portable CT scanner in an intensive care unit room and 4 slices from a non-contrast pCTH examination.

In addition to fulfilling the European Union standards for medical devices, to fulfil the French ethical and safety standards, the study obtained authorizations from the national ethics review board of Ile-de-France (No. 018-085, ESPER, ID-RCB: 2018-A02723-52) and from the French Nuclear Security Agency to use the portable CT scanner Ceretom (NeuroLogica Corporation, Samsung Electronics Co. Ltd, Danvers, Massachusetts, USA) (Fig. 3)^12^. All methods were performed in accordance with the Declaration of Helsinki. The pCTH device was stored in the ICU. cCTH was performed with an Aquilion Prime TSX-303A CT scanner, Toshiba (Tustin, California, USA) and was located on the floor just below the ICU, with a maximum transfer distance of 140 m for the ICU bed furthest from the cCTH device. The radiographers underwent a training period before the pCTH device was deployed in the ICU.

Adult patients (> 18 years old) admitted to the ICU for severe acute brain injury (Glasgow Coma Scale score < 9), including traumatic brain injury (TBI), aneurysmal subarachnoid haemorrhage (aSAH), spontaneous intracranial haemorrhage (sICH) and ischaemic stroke, who had invasive intracranial pressure monitoring and who required a single CTH scan in the first 7 days following ICU admission were eligible. Patients were excluded if they were pregnant or expected to live < 24 h. Despite the COVID-19 pandemic period, the management of and procedures for performing cCTH in ICU patients did not change. Due to the organization of the imaging department and to the availability of radiographers, we initially define a time slot for pCTH performance from 1:00 pm to 5:00 pm during working days. cCTH could be performed at any time of day. The management of TBI and aSAH in the ICU followed the international guidelines for ICUs^1,13^.

The primary outcome was to compare the time required to perform a CTH scan. We hypothesized that pCTH procedure lasts shorter than cCTH. The duration of each scanning procedure, defined as the time from the beginning of the preparation for each procedure to its end (all monitoring devices and ventilator reconnected, and the patient back in his/her position before the procedure), was recorded. For pCTH, this duration started from the time the radiographer moved the pCTH to the patient ICU bedroom to the end of procedure (patient back in his/her position before the procedure). This latter duration definition aims to consider the delay required to perform the whole procedure (work of radiographer staff which may impact the length of the procedure) to make fair comparison between the 2 CTH modalities.

### Collected data

Demographic and clinical data, including age, sex, reason for admission, and GCS score upon admission, were collected. The reason for performing a CTH scan was noted. During CTH, clinical parameters were recorded every minute, including mean arterial pressure (MAP), intracranial pressure and cerebral perfusion pressure. Intracranial pressure was monitored via intraparenchymal fibre or external ventricular drain (EVD). Adverse reaction was defined according the ICH guidelines (ICH GCP E6(R2), https://database.ich.org/sites/default/files/E6_R2_Addendum.pdf). Their occurrence and their duration during the CTH procedure and/or IHT for CTH were reported, including arterial hypotension (systolic arterial blood pressure < 110 mmHg), intracranial hypertension (ICP ≥ 20 mmHg) and the use of osmotherapy if needed, increases in the infusion rates of norepinephrine, changes in ventilator settings, ventilator disconnection, cardiac arrythmias, episode of oxygen saturation < 95%, and arterial-line or central-line disconnection. We categorized patients as having a high risk of transport-related adverse reactions if they were physiologically unstable with cardiovascular instability (requirements of vasopressors), required mechanical ventilation with FiO2 > 50%, or had intracranial hypertension (ICP ≥ 20 mmHg). Medium-risk patients were categorized as those who were physiologically stable without vasopressors under mechanical ventilation (FiO2 < 50%) and without intracranial hypertension. All other patients represented low-risk patients. CTH hardware or software failures were also reported, and in case of failure or poor quality images, additional cCTH were identified in pCTH group. The number of medical and paramedical staff involved, including medical doctors, nurses, caregivers and radiographers, was identified for each procedure.

The occurrence of nosocomial infection during the ICU stay was also recorded. Additionally, the in-ICU mortality rate and the neurological outcome at 3 months were assessed using the Glasgow Outcome Scale through a telephone interview by a trained clinical research assistant.

### Statistical analysis

Based on real measurements of moving a patient from an ICU-bed to the imaging room in our centre, the median time (± standard deviation (SD)) required to perform a cCTH scan was estimated to be 35 ± 4 min, including intrahospital transport, cCTH performance and patient’s installation back in the ICU bed. The transport phase represented approximately 16 min back and forth (unpublished personal data). We calculated that a sample size of at least 36 patients (18 in each group) would be necessary to detect a reduction of 15 min in CTH performance when using pCTH, with a standard deviation of 8 min, a type I error of 5% and a power of 90%. To compare serious adverse reactions such as ventilator disconnection, we estimated the sample size to be at least 190 patients (95 patients in each group for a type I error of 5% and a power of 90%). For mABP, ICP and CPP comparisons between groups, we performed a non-parametric Kruskal–Wallis–Wilcoxon test at each time point and applied a Bonferroni correction to address the multiple comparisons. Additionally, for graphical representation of duration of CTH overtime, a linear regression was performed in each group. Data were analysed using R Core Team (2020). R: A language and environment for statistical computing. R Foundation for Statistical Computing, Vienna, Austria. (https://www.R-project.org/). Quantitative variables are expressed as the median (interquartile), and nonparametric Mann–Whitney tests were used if required. Qualitative variables are expressed as n (%), and data were compared using the nonparametric Kruskal–Wallis–Wilcoxon test. For quantitative data such mABP, ICP and CPP, comparisons at each time point were performed with the nonparametric Kruskal–Wallis–Wilcoxon test using a Bonferroni posthoc correction to address multiple comparisons. For all analyses, a p value < 0.05 was considered significant.

### Ethics approval and consent to participate

The study obtained authorizations from the national ethics review board of Ile-de-France (No. 018-085, ESPER, ID-RCB: 2018-A02723-52) and from the French Nuclear Security Agency to use the portable CT scanner Ceretom. Written informed consent was obtained from all the patients or patient relatives.

## Data Availability

The datasets used and/or analysed during the current study are available from the corresponding author on reasonable request.

## Abbreviations

ABI: Acute brain injury
aSAH: Aneurysmal subarachnoid haemorrhage
cCTH: Conventional computed tomography of head
COVID-19: Coronovirus disease-19
CPP: Cerebral perfusion pressure
GCS: Glasgow coma scale
ICH: Intracranial hypertension
ICP: Intracranial pressure
ICU: Intensive care unit
IHT: Intra-hospital transfer
MAP: Mean arterial pressure
pCTH: Portable computed tomography of head
TBI: Traumatic brain injury
